# When a foreign gene meets its native counterpart: computational biophysics analysis of two *PgiC* loci in the grass *Festuca ovina*

**DOI:** 10.1038/s41598-020-75650-0

**Published:** 2020-10-30

**Authors:** Yuan Li, Sandipan Mohanty, Daniel Nilsson, Bengt Hansson, Kangshan Mao, Anders Irbäck

**Affiliations:** 1 Computational Biology and Biological Physics, Department of Astronomy and Theoretical Physics, Lund University, 223 62 Lund, Sweden; 2 Institute for Advanced Simulation, Jülich Supercomputing Centre, Forschungszentrum Jülich, 52425 Jülich, Germany; 3 Department of Biology, Lund University, 223 62 Lund, Sweden; 4 Key Laboratory of Bio-Resource and Eco-Environment of Ministry of Education, College of Life Sciences, State Key Laboratory of Hydraulics and Mountain River Engineering, Sichuan University, Chengdu, 610065 China

**Keywords:** Biophysics, Evolution

## Abstract

Duplicative horizontal gene transfer may bring two previously separated homologous genes together, which may raise questions about the interplay between the gene products. One such gene pair is the “native” *PgiC1* and “foreign” *PgiC2* in the perennial grass *Festuca ovina*. Both *PgiC1* and *PgiC2* encode cytosolic phosphoglucose isomerase, a dimeric enzyme whose proper binding is functionally essential. Here, we use biophysical simulations to explore the inter-monomer binding of the two homodimers and the heterodimer that can be produced by *PgiC1* and *PgiC2* in *F. ovina*. Using simulated native-state ensembles, we examine the structural properties and binding tightness of the dimers. In addition, we investigate their ability to withstand dissociation when pulled by a force. Our results suggest that the inter-monomer binding is tighter in the PgiC2 than the PgiC1 homodimer, which could explain the more frequent occurrence of the foreign PgiC2 homodimer in dry habitats. We further find that the PgiC1 and PgiC2 monomers are compatible with heterodimer formation; the computed binding tightness is comparable to that of the PgiC1 homodimer. Enhanced homodimer stability and capability of heterodimer formation with PgiC1 are properties of PgiC2 that may contribute to the retaining of the otherwise redundant *PgiC2* gene.

## Introduction

Horizontal gene transfer (HGT) is a phenomenon in which organisms acquire genetic material from a different species, via unconventional means (e.g. via vectors such as a shared virus^1^) rather than hybridization and reproduction^2^. Among non-parasitic flowering plants, HGT of functional nuclear genes has been shown to be uncommon^3^, but frequent in grasses^4–6^, one example being the *PgiC2* gene in the perennial grass *Festuca ovina*^4,7,8^. *PgiC2* has been suggested to be horizontally transferred into *F. ovina* from another grass genus *Poa*, based on the incongruent phylogenetic relationship between *PgiC2* and the corresponding genes in several different *Festuca* species^4,7^ as well as the close sequence similarity between the down- and upstream (containing a transposition associated factor) regions of *PgiC2* and those of the corresponding gene in one potential *Poa* donor species^8^. The horizontal transfer of *PgiC2* from *Poa* to *F. ovina* has been estimated to have occurred ca.<600000 years ago^4^.

Most of the horizontally transferred functional nuclear genes between grasses identified so far (including *F. ovina* *PgiC2*) have native counterparts in the recipient species (i.e. duplicative HGT)^5^. The fate of a gene acquired via duplicative HGT is like that of a gene duplicate^9^, which could be nonfunctionalization^10^, neofunctionalization^11^, subfunctionalization^12^, or redundancy^13^. The last possibility may be immediately beneficial when the native gene copy accidentally loses its function due to reasons like lethal mutations^9^. In the grass *F. ovina*, the predominant *PgiC2* gene product has been shown to more frequently occur in dry habitat^3^, implying that the foreign *PgiC2* gene products may have some superior properties (over the native *PgiC1* gene products) that hold the potential to make its presence beneficial to *F. ovina* under drought stress. Thus our first goal in the current study is to look for such properties.

In addition, both the *F. ovina* *PgiC2* gene and its native counterpart *PgiC1* code for the cytosolic phosphoglucose isomerase (PgiC) enzyme, which is a metabolic enzyme that catalyses the reversible isomerization between glucose-6-phosphate and fructose-6-phosphate in glycolysis at a metabolic intersection spot that is also connected with several other metabolic pathways, e.g. starch/sucrose synthesis and gluconeogenesis^14–16^. The functional unit of a PgiC protein is a dimer formed by two polypeptides^17^, and the catalytic centres of a PgiC protein are composed of residues contributed by both monomers. Therefore, the proper association of the two monomers within a dimeric PgiC protein is essential not only for structural stability but also for the function^18^. In *F. ovina*, it has been shown that, in addition to the PgiC2 homodimer, the polypeptide product of *PgiC2* is able to form a PgiC1–PgiC2 heterodimer with the polypeptide encoded by *PgiC1*^3,19^. Therefore, the second goal of the current study is to investigate how the two genes, originally separated by speciation, structurally “get along” with each other when forming a heterodimer at their abrupt encounter after the duplicative HGT.

In this paper, to address these goals, we present a Monte Carlo (MC)-based computational investigation of all these three PgiC dimers in *F. ovina*. Using reference dimer structures based on homology modelling, two sets of biophysical MC simulations were conducted. The aim of the first set was to explore native-state conformational fluctuations of the dimers. Based on the resulting native-state ensembles, structural and inter-monomer binding properties of the dimers were investigated. The second set of simulations explored the mechanical response of the dimers to a stretching force. The ability of the dimers to withstand dissociation when pulled by a force depends on the inter-monomer binding tightness. We find that these two different approaches lead to very similar conclusions regarding the relative binding tightnesses among the three dimers. Both methods suggest that the binding is tighter in the PgiC2 than the PgiC1 homodimer, and the binding strength of the PgiC1–PgiC2 heterodimer is comparable to that of the native PgiC1 homodimer.

There have been many previous molecular simulation-based studies of the role of dimer stability in biological processes^20–23^. Compared to previously studied systems, the PgiC dimer is unusually large, with >500 residues per monomer unit.

## Results

### Protein sequences and predicted dimer structures

We selected one representative *F. ovina* PgiC1 sequence (GenBank accession no. AED99454) and one representative *F. ovina* PgiC2 sequence (AED99455) for our study. These protein sequences both contain 567 residues, and differ at 20 of the residue positions (48, 49, 53, 62, 85, 109, 118, 121, 123, 200, 210, 237, 266, 312, 318, 372, 455, 466, 521, 554; see Supplementary Table S1). With these two sequences as input, we predicted 3-D native structures of the three protein systems studied: the PgiC1 and PgiC2 homodimers and the PgiC1–PgiC2 heterodimer. For this task, we used homology modelling, supplemented with protein docking in the heterodimer case (see “Methods”). Throughout the rest of the paper, we restrict ourselves to the 549 residue long 6–554 segments of the full-length sequences, due to poor alignment between the target and template sequences in the N- and C-termini. A close examination of the 20 variable residue positions in the modelled 3-D structures revealed no major structural differences between the three protein systems around these residues, for instance, with respect to secondary structure (Supplementary Table S1, Supplementary Figs. S1 and S2). Four of the variable residue positions are near the dimer interface (200, 372, 466, 521).

The potential functional impact of mutating PgiC1 to PgiC2 residues at the variable sites was tested using the SNAP2 program^24–26^. No strong signal of functional impact was obtained, but a weak signal of functional effect was found at six of the 20 positions (109, 118, 237, 266, 372, 554; see Supplementary Table S1).

### Native-state properties from simulated ensembles

In their native state, proteins are not completely rigid but undergo structural fluctuations. To characterise the native states of our three PgiC dimers, we therefore generated ensembles of native-like structures through extensive MC simulations (see “Methods”), starting with the predicted structures described above as initial structures. In the simulations, a constraint was imposed on the monomer structures, by penalising large root-mean-square deviations (RMSDs) from the initial monomer structures (see “Methods”). With this constraint in place, but no inter-monomer constraint imposed, the dimers stayed intact throughout the runs. To gather statistics, a set of 24 independent runs was generated for each dimer, and 3000 snapshots were stored in each run. A Supplementary Video illustrates the MC evolution in a typical (part of a) run. Based on these simulated conformational ensembles, we analyzed the secondary structure, the solvent-accessible surface area (SASA) and the inter-monomer interactions of the dimers. All properties were evaluated conformation by conformation, and then averaged over the simulated ensembles.

The secondary structure of the dimers is mixed, with a large amount of helical structure and some strand structure (Supplementary Fig. S1). A residue-wise secondary-structure analysis was carried out using STRIDE^27^ assignments. Only minor differences were detected between the three dimers. The global helix content was found to be 50.1–50.4% for the three systems. The global strand content was 12.0% for the PgiC1–PgiC2 heterodimer and marginally lower, 11.8%, for the two homodimers (ANOVA test: $p = 0.01$, Tukey’s range test: $p = 0.03/0.02$)^28^. These results indicate that the three native dimers are very similar in terms of secondary-structure content, although the exact numbers may be affected by the enforced constraint on global RMSD.

**Figure 1:**
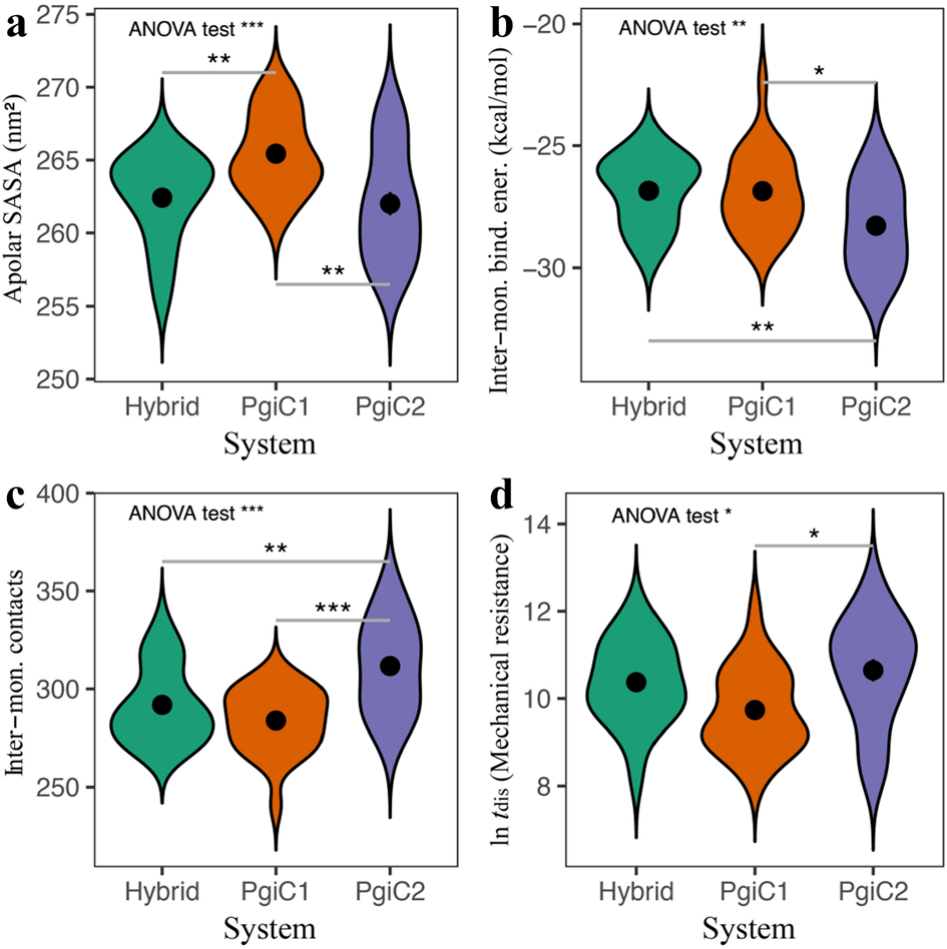
Basic biophysical properties of the three dimers. The four panels show (**a**) apolar SASA, (**b**) inter-monomer binding free energy, (**c**) the number of inter-monomer residue-pair contacts, and (**d**) the mechanical resistance, as measured by the logarithm of the dimer dissociation time, $$\ln t_\text {dis}$$, in MC pulling simulations. The data in (**a**–**c**) are based on the simulated native-state ensembles, whereas the data in (**d**) come from the pulling simulations (see “Methods”). Standard errors are comparable in size to the plot symbols. Violin plots illustrate how the raw data are distributed. Asterisks indicate significance levels ($$*$$: $$0.01< p < 0.05$$, $$**$$: $$0.001< p < 0.01$$, $$***$$: $$p < 0.001$$).

Despite this overall similarity, differences may still exist in local secondary-structure propensities along the chains; the small differences in overall secondary-structure content may be localised to particular chain regions rather than being uniformly spread over the entire polypeptide chains. Therefore, we also computed and compared residue-specific secondary-structure profiles for the three dimers (Supplementary Fig. S2). The comparison reveals that the difference between the three systems in general is small, but some potential exceptions exist. To precisely assess the significance of these potential differences would require more statistics. Nevertheless, we note that there are four residue positions (364, 377, 418, 425), all of which sit on or near the dimer interface, where the $\beta$-strand propensity is notably higher in the PgiC2 homodimer than in the other two dimers (Supplementary Fig. S2). Another important characteristic of the dimers is their solvent-accessible surface area (SASA), which we evaluated using the FreeSASA program^29^. As can be seen from Fig. 1a, the apolar SASA of the PgiC1 homodimer (265.4 nm$^2$) is larger than those of the heterodimer (262.4 nm$^2$) and the PgiC2 homodimer (262.0 nm$^2$). Moreover, statistical tests suggest that these differences, unlike those in secondary structure discussed above, are highly significant (ANOVA: $p = 7.1\times 10^{-4}$, Tukey: $p=5.2\times 10^{-3}/1.3\times 10^{-3}$). However, the small difference between the heterodimer and the PgiC2 homodimer is not significant. This analysis was repeated for the total SASA, including polar SASA as well, with similar results.

The catalytic centre of the PgiC dimer is composed of residues from both monomers, which suggests that proper binding of the two monomers is essential not only structurally but also functionally. To get a measure of the inter-monomer binding strength, we estimated the binding free energy, $\Delta G$, of the dimers using the PRODIGY program^30^. Averaging over the native-state ensembles, we obtained $\Delta G=-28.3$ kcal/mol for the PgiC2 homodimer, $\Delta G=-26.9$ kcal/mol for the PgiC1 homodimer, and $\Delta G=-26.8$ kcal/mol for the heterodimer (Fig. 1b). Statistically, the $\Delta G$ value is significantly lower for the PgiC2 homodimer than for the other two dimers (ANOVA: $p = 3.7\times 10^{-3}$, Tukey: $p = 9.5\times 10^{-3}/0.01$). A simpler measure of the amount of inter-monomer interaction is the number of inter-monomer residue-pair contacts, $n_\text {c}$. A residue pair is said to be in contact if there is any inter-residue pair of heavy atoms within 5.5 Å from each other. In line with the above $\Delta G$ results, this number turned out to be largest for the PgiC2 homodimer with $n_\text {c}=311.7$. The corresponding numbers for the PgiC1 homodimer and the heterodimer were $n_\text {c}=283.8$ and $n_\text {c}=291.8$, respectively (Fig. 1c). Again, the differences between the PgiC2 homodimer and the other two dimers were statistically significant (ANOVA: $p = 2.9\times 10^{-5}$, Tukey: $p = 3.0\times 10^{-3}/2.6\times 10^{-5}$). Finally, we also performed a direct calculation of the average binding energy, using the energy function on which our simulations are based^31^. In perfect agreement with the contact analysis, the binding energy was lower for the PgiC2 homodimer ($-83.8$ kcal/mol) than for the PgiC1 homodimer ($-73.5$ kcal/mol) and the heterodimer ($-76.1$ kcal/mol). The data for these different quantities, thus, consistently suggest a stronger binding for the PgiC2 homodimer than for the other two dimers.

**Figure 2:**
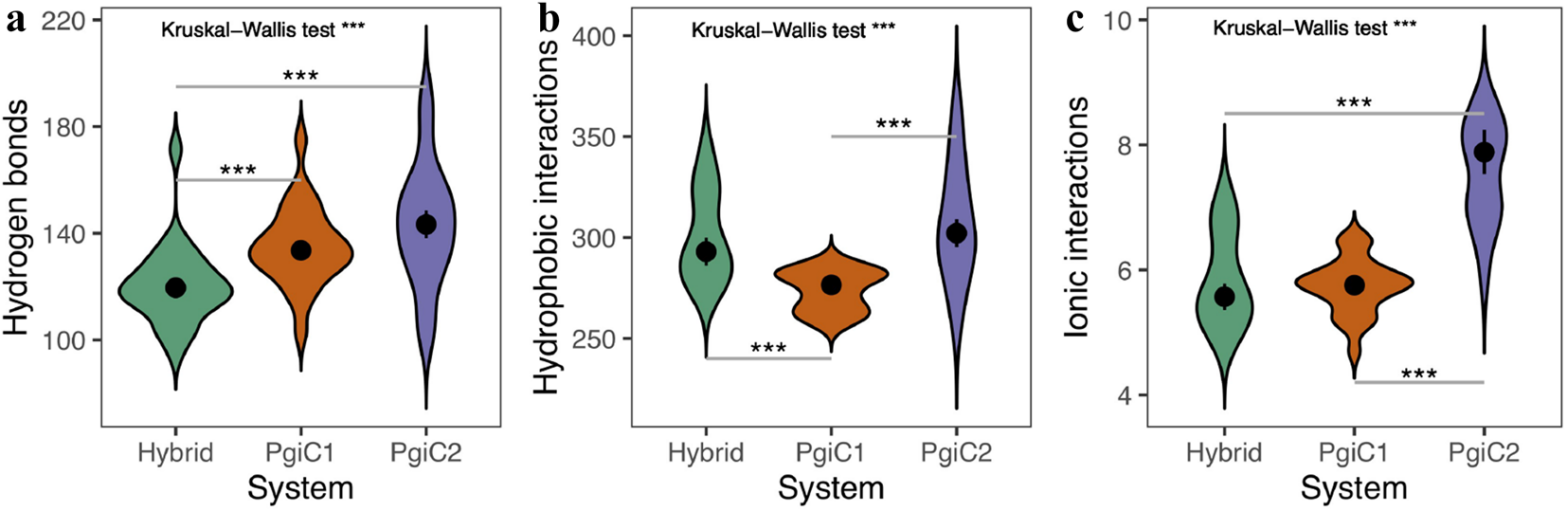
Abundance of different types of inter-monomer interaction in the three dimers. The three panels show (**a**) the number of hydrogen bonds, (**b**) the number of interactions between hydrophobic groups, and (**c**) the number of ionic interaction. Plot symbols indicate medians. Standard errors, from 10,000 bootstrap repeats, are indicated. Violin plots illustrate the distribution of data points. The data for different dimers were compared by a Kruskal–Wallis test followed by pairwise Wilcoxon signed ranks tests (with correction for multiple testing). Asterisks indicate level of significance ($$***$$: $$p < 0.001$$).

To investigate what forces are responsible for the binding affinity of the dimers, we examined the relative abundance of three major types of inter-monomer interaction, using the YASARA program^32^. Specifically, focusing on interactions across the dimer interface, we counted the number of hydrogen bonds, the number of interactions between hydrophobic groups, and the number of ionic interactions (Fig. 2). Cation-pi interactions were counted as well, but found to be rare (on average between 1.8 and 2.5 interactions). Most abundant was the hydrophobic form of contact (276–302 interactions). A comparison of the dimers shows that all the three interaction types examined were more abundant in the PgiC2 homodimer than in the other two systems, though the differences are not in all cases significant (Fig. 2).

**Figure 3:**
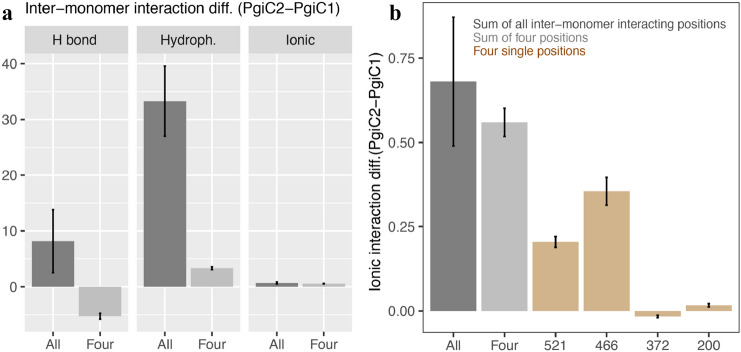
Contribution of the four variable residue positions at the dimer interface (200, 372, 466, 521) to inter-monomer interactions in the PgiC1 and PgiC2 homodimers. Differences in the number of inter-monomer interactions of a given type between the PgiC2 and PgiC1 homodimers. (**a**) Differences obtained when considering, respectively, all inter-monomer interactions and those that involve a variable position (200, 372, 466 or 521). The latter type of interactions does not explain the observed overall difference between the two dimers in hydrogen bonds or hydrophobic interactions. (**b**) The overall difference in ionic inter-monomer interactions between the two dimers can, by contrast, be largely linked to the four variable positions. In fact, a closer analysis of the individual contributions of these four positions reveals that two of them, 466 and 521, are responsible for a major part of the overall difference.

A natural question is to what extent the higher number of inter-monomer interactions in the PgiC2 homodimer can be directly traced to the four variable residue positions near the dimer interface (200, 372, 466, 521). Therefore, focusing on the two homodimers, we also counted inter-monomer interactions involving these four specific positions. It turns out that these positions explain very little of the overall differences between the PgiC1 and PgiC2 homodimers in inter-monomer hydrogen bonds and hydrophobic interactions, respectively (Fig. 3a). By contrast, for the ionic type of interaction, the subset of interactions involving these positions accounts for >80% of the overall difference between the two dimers (Fig. 3a,b). Furthermore, a closer analysis reveals that this major contribution from the four variable positions can be entirely attributed to only two of them (466 and 521), while the contribution from the other two positions (200 and 372) is negligible (Fig. 3b).

Four other interesting residue positions that sit on or near the dimer interface are those identified above (364, 377, 418, 425) at which the PgiC2 homodimer displays a notably higher $\beta$-strand content than the other two proteins. A similar analysis of these positions gave, however, no indication that the stronger inter-monomer interaction propensity of the PgiC2 homodimer can be linked to these positions.

In summary, the above analysis of the binding strength of the dimers, based on our simulated native-state ensembles, suggests that the binding is strongest for the PgiC2 homodimer. Hydrogen bonding, hydrophobic interactions and ionic interactions between the monomers were all found to be more abundant in the PgiC2 homodimer than in the other two dimers. These differences, in general, cannot be attributed to a small set of specific residue positions. However, the more abundant ionic interactions of the PgiC2 homodimer are largely explained by two variable residue positions located at the dimer interface.

### Mechanical resistance in pulling simulations

Another computational approach that can shed light on the binding tightness of a dimer is by pulling simulations, where the response to external mechanical forces is probed^20,22^. Taking this approach, we simulated the three PgiC dimers when subject to equal and oppositely directed forces acting on the $\hbox {C}_\alpha$ atoms closest to the centers of mass of the respective monomers. The force strength was held constant at a value (368 pN) chosen such that the dimers stayed intact over a significant period of time, while still dissociating within a computationally manageable time. For each of the three dimers, a set of 24 independent pulling simulation runs was conducted, all started with the dimer in its native state.

**Figure 4:**
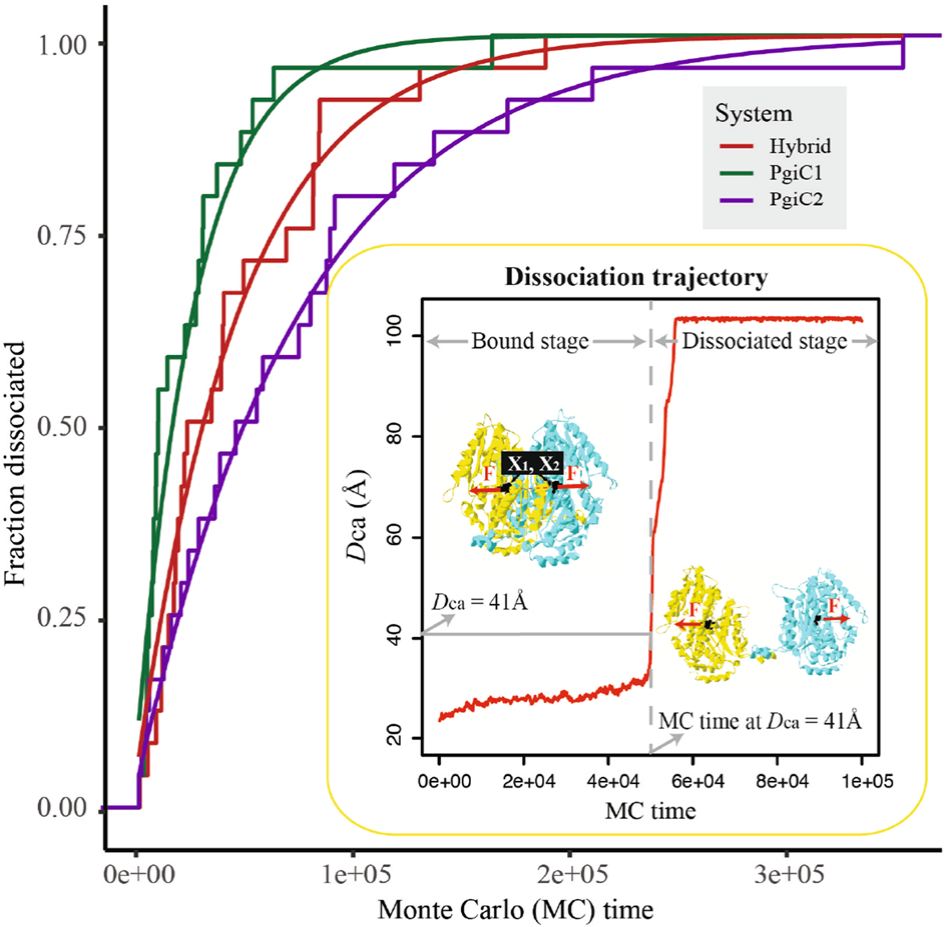
Dimer dissociation in MC pulling simulations. In these simulations, started from the native state, the dimer is subject to a constant stretching force (368 pN), which acts on two $\hbox {C}_\alpha$ atoms located near the centers of mass of the respective monomers. For each of the three dimers, a set of 24 independent runs was generated. The figure shows the fraction of runs in which the dimer is in a dissociated state, *P*(*t*), as function of MC time, *t*, for the PgiC1 homodimer (green), the heterodimer (red) and the PgiC2 homodimer (purple). The smooth curves show the expected behavior for a simple two-state dissociation process, $P(t)=1-\text {e}^{-\lambda t}$, where the parameter $\lambda$ was computed as the inverse mean dissociation time for a given dimer, rather than by fitting to *P*(*t*) data. The inset shows the MC evolution of the distance between the two central $\hbox {C}_\alpha$ atoms, $D_\text {ca}$, in a representative run. The dimer stays native-like over a significant period of MC time, followed by a sudden dissociation event signalled by a rapid increase in $D_\text {ca}$. Dissociation is said to occur when $D_\text {ca}$ passes a threshold, set to 41 Å.

The inset in Fig. 4 shows the MC evolution of the distance between the two central $\hbox {C}_\alpha$ atoms, $D_\text {ca}$, in a representative run. In the initial phase of the run, the $D_\text {ca}$ value stays low, indicating that the dimer remains native-like. This phase ends by a sudden and large increase in $D_\text {ca}$, signalling the dissociation of the dimer. The fact that the $D_\text {ca}$ levels off (at about 103 Å) after the dissociation event is due to the finite size of the simulation box. The dissociation is a stochastic event, so the MC time elapsed before dissociation occurs varies from run to run. For a given run, the dissociation time, $t_\text {dis}$, may be defined as the time at which the $D_\text {ca}$ passes a threshold, which we take to be 41 Å. This threshold value is sufficiently large to ensure that, in our runs, once the system has passed the threshold, it never returns to lower $D_\text {ca}$ values.

The dissociation time, $t_\text {dis}$, provides a measure of the mechanical resistance of the dimer. Averaging over the 24 runs for each system, a dissociation time of $t_\text {dis}=4.2\times 10^4$ MC cycles was obtained for the mechanically most resistant dimer, the PgiC2 homodimer. The corresponding values were $t_\text {dis}=3.2\times 10^4$ MC cycles for the heterodimer and $t_\text {dis}=1.7\times 10^4$ MC cycles for the PgiC1 homodimer. Fig. 1d shows data for the logarithm of the dissociation time. Statistical tests on the $\ln t_\text {dis}$ data suggest that the mechanical resistance of the PgiC2 homodimer indeed is significantly higher than that of the PgiC1 homodimer (ANOVA: $p = 0.01$, Tukey: $p = 0.01$).

Alternatively, the mechanical resistance of the dimers can be quantified by computing the fraction of runs, *P*(*t*), in which the dimer has dissociated at a given MC time *t*. The *P*(*t*) data, plotted against *t*, support the above conclusion that the dissociation occurs more rapidly for the PgiC1 homodimer than for the PgiC2 homodimer (Fig. 4). Again, the data for the heterodimer fall in between those for the two homodimers. For all three dimers, the *P*(*t*) data are quite well described by an exponential saturation curve, $P(t)=1-\text {e}^{-\lambda t}$, which is the behaviour one would expect for a simple two-state dissociation process (Fig. 4). In drawing these curves, the parameter $\lambda$ was not fitted to data, but rather determined as the inverse of the average dissociation time, $\lambda =1/t_\text {dis}$.

The relatively strong binding tightness of the PgiC2 homodimer in the native-state simulations described earlier matches very well with the enhanced mechanical resistance of this dimer seen in the pulling simulations.

## Discussion

This study focuses on one foreign gene (*PgiC2*) and its native counterpart (*PgiC1*) in the grass *F. ovina*^4,7,8^. Both genes encode a PgiC protein, the functional unit of which is a dimer comprising two homological monomers. The catalytic centres of a PgiC protein are composed of residues contributed by both monomers^18^. Therefore, the proper association of the two monomers within a PgiC protein is essential not only for PgiC’s structural stability but also for its function^18^.

In this study, we have modelled the 3-D structures of the three types of PgiC proteins that are produced by the above-mentioned foreign-native gene pair: the PgiC1 homodimer (encoded entirely by the native *PgiC1* gene), the PgiC2 homodimer (coded for completely by the foreign *PgiC2* gene) and the PgiC1–PgiC2 heterodimer. Starting from these modelled structures, we conducted biophysical simulations of the three dimers, to explore their native-state conformational ensembles and their mechanical resistance when pulled by an external force. All simulations were carried out under a constraint on the global structure of the monomers, to ensure that the monomers stayed native-like throughout the runs. No constraints were imposed on the relative position or orientation of the monomers in the simulated dimers. While artificial, this constraint still permits local structural fluctuations to occur anywhere along the polypeptide chain. Therefore, the generated conformational ensembles should offer a less biased understanding of the biophysical properties of the dimers, compared to examining single rigid structures.

The mechanical resistance shown by the dimers in the pulling simulations correlates well with their binding free energy as estimated from the native-state simulations. Both methods suggest a tighter inter-monomer binding in the PgiC2 than the PgiC1 homodimer and the hybrid heterodimer (Fig. 1b,d). This picture is further supported by comparing the abundance of inter-monomer residue-pair contacts in the three dimers (Figs. 1c and 2). Furthermore, analysis of the solvent-accessible surface area yielded a lower apolar SASA for the PgiC2 homodimer than for the other two dimers (Fig. 1a). At the same time, the amount of hydrophobic (apolar) inter-monomer contacts (Fig. 2b) was found to be higher for the PgiC2 than the PgiC1 homodimer. These findings all support the view that among the three studied dimers the PgiC2 homodimer is most stable. We expect this conclusion to be insensitive to small changes in temperature. However, it should be noted that, due to the computationally challenging size of the systems studied, our investigation focused on a single temperature (300 K).

An interesting question is whether the differences in binding tightness between the three dimers can be traced to specific residues. The computed residue-wise secondary-structure profiles were very similar (Supplementary Fig. S2), but indicated an elevated $\beta$-strand propensity for the PgiC2 homodimer at four positions (364, 377, 418, 425), all located at or near the dimer interface. However, a residue-pair contact analysis did not reveal any noticeable differences among the dimers in the abundance of inter-monomer contacts involving these four positions (data not shown). At these four residue positions, the PgiC1 and PgiC2 sequences are identical. Among the 20 identified variable positions, four are located near the dimer interface (200, 372, 466, 521)^33,34^, and therefore of special interest (Supplementary Table S1, Supplementary Fig. S1). Two of these sites (466 and 521), at both of which PgiC2 unlike PgiC1 has a charged residue, turned out to account for >80% of the difference between the PgiC1 and PgiC2 homodimers in ionic inter-monomer contacts (Fig. 3b). However, the contact analysis also indicates (Fig. 3) that the main difference between the homodimers is in hydrophobic rather than ionic inter-monomer contacts. Therefore, except for a limited contribution from changes in the electrostatics at sites 466 and 521, it seems that the difference in inter-monomer binding strength between the PgiC1 and PgiC2 homodimers is mainly due to indirect conformational effects, rather than to direct effects of residue substitutions.

Could the presence of a more stable foreign gene product have any ecological significance? In plants, severe stresses usually induce the production of protein-stabilizing molecules (such as dehydrins and chaperones)^35,36^, indicating that stress-induced protein denaturation is common. This is probably what happens to the PgiC1 homodimer under extreme water deficit conditions in the Alvar grassland on Öland (Sweden), where the ecological genetic studies of the *F. ovina* PgiC proteins were carried out^3,37^. This biological environment is characterised by a thin layer of (or no) soil on a limestone plain, so the soil in some area there can easily dry up during summer, leading to intense desiccation. A loss of function of the PgiC1 homodimer may be especially problematic when stress-induced, because the glycolysis pathway that the PgiC protein is involved in plays an important role in providing both ATP and biosynthetic precursors for stress acclimation^38^.

In such adverse circumstances, the presence of a more stable PgiC2 homodimer that is more likely to stand the stress and stay functional may compensate for the denaturation-induced functional loss of the PgiC1 homodimer. This could be why the presence of the principal *PgiC2* gene product has been found to be significantly associated with dry environmental conditions in the Alvar populations of *F. ovina* on Öland^3^.

The occurrence of a functional heteromer between a paralogous gene pair (like the PgiC1–PgiC2 heterodimer), may lead to a situation where the two genes require each other’s presence for maintaining their functions^39^. This is, however, not likely to be the case in the current system, because the foreign *PgiC2* gene is not fixed in the *F. ovina* populations (at least not yet). Instead, it can only be found in some individuals^40^, and even when it does occur, its presence can be very complex and cryptic. For example, silenced *PgiC2* (pseudogene) is very common for individuals with *PgiC2*^40^, and the *PgiC2* gene may also exist as tandem duplicate and/or hemizygote^19,41^. All of these facts may suggest that under normal rather than extreme water-limited conditions, the presence of the foreign *PgiC2* gene is inessential.

Should one expect the products of the two previously separated *PgiC1* and *PgiC2* genes to be capable of heterodimer formation? Beside the cytoplasmic PgiC protein, plants also have a plastid-specific phosphoglucose isomerase, hereafter referred to as PgiP, which has been suggested to have a bacterial origin (maybe from cyanobacteria)^42,43^. Consistent with this, plant PgiP has a much higher protein sequence identity with cyanobacteria PgiC (*Arabidopsis thaliana* PgiP [Q8H103]–*Cyanobacterium aponinum* PgiC [K9Z8L2]: 62%) than with plant PgiC (*A. thaliana* PgiP [Q8H103]–*A. thaliana* PgiC [BAB17653]: 30%). From the low protein sequence similarity between PgiP and PgiC in plants, we can see that these two phosphoglucose isomerases diverged very long ago. As a result, lots of differences have accumulated between them at the dimer interface, so that no heterodimer can be formed between them anymore^44^.

In the *F. ovina* *PgiC1*-*PgiC2* system, the two previously separated genes are suddenly brought together by one or more recent inter-genus HGT events between *F. ovina* and a *Poa* species^4,8^. In contrast to the PgiC-PgiP pair, the PgiC1 and PgiC2 polypeptides can still form a heterodimer that functions properly^3,19^. The results presented here indicate an inter-monomer binding that is at least as strong in the PgiC1–PgiC2 heterodimer as it is in the native PgiC1 homodimer. This may not be surprising considering that PgiC heterodimers can form even between monomers from different plant families (e.g., between spinach [Amaranthaceae] and *Clarkia* [Onagraceae] PgiC, between cauliflower [Brassicaceae] and *Clarkia* PgiC, and between cauliflower and sunflower [Asteraceae] PgiC)^44^. In addition, inter-family PgiP heterodimers are possible (e.g., between cauliflower and sunflower PgiP, and between sunflower and spinach PgiP)^42^. That all these inter-taxon PgiC or PgiP heterodimers can occur probably reflects the essential functions of the PgiC or PgiP proteins. The need to ensure their proper function may explain why their 3-D structures are relatively conserved among (at least not too distant) organisms (see e.g. the 3-D structures of the *Francisella tularensis* bacterium, pig, and *Colias* butterfly PgiC, with PDB codes 3LJK^45^, 1GZD^46^ and 4WMJ^47^).

### Conclusion

The *F. ovina* *PgiC2* gene represents an interesting example of exchanges of functional nuclear genes between distantly related non-parasitic flowering plant species. Ecological significance has been suggested for the presence of this foreign *PgiC2* gene in *F. ovina* under stressful environmental conditions on Öland, Sweden^3^. In support of this suggestion, our computational analysis has found a significantly stronger inter-monomer binding for the PgiC2 homodimer than for the PgiC1 homodimer, suggesting that a higher stability of PgiC2 (than PgiC1) that may help the host handle extreme stresses. This finding is supported by evidence derived both from equilibrium simulations of the native dimers, and from simulations probing the ability of the dimers to withstand dissociation when pulled by an external force. A second aim of this study was to find out how the foreign *PgiC2* gene and its native counterpart *PgiC1* get along with each other when forming a heterodimeric protein product, at their abrupt encounter after the horizontal gene transfer event. The PgiC1–PgiC2 heterodimer was found to show inter-monomer binding properties consistent with a proper functioning of the hybrid protein, with a binding strength comparable to that of the native PgiC1 homodimer. The results presented here thus support a picture where the foreign *PgiC2* gene in *F. ovina* is conditionally advantageous over the native *PgiC1* gene, and at the same time able to “collaborate” with the native gene in a non-harmful manner (through the formation of a hybrid protein). These factors may contribute to the retaining of this otherwise redundant foreign gene.

## Methods

### Dimer structure prediction

Representative protein sequences for *F. ovina* PgiC1 (GenBank accession no. AED99454) and PgiC2 (AED99455) were identified and downloaded from the GenBank database (the only two full-length sequences available). Differences between the two sequences are summarised in Supplementary Table S1. The 3-D structures of the PgiC1 and PgiC2 homodimers were then predicted by homology modelling using the SWISS-MODEL software^48,49^. The crystal structure of *Toxoplasma gondii* PgiC (PDB code 3UJH.1, bound with G6P) served as template. This template has a high sequence identity with the input PgiC1 and PgiC2 sequences (56% and 55%, respectively). The modelled structures were relaxed using Rosetta (v. 3.7) all-atom refinement^50^, to relieve steric clashes. For the PgiC1–PgiC2 heterodimer, a rigid-body docking strategy was adopted, using the ZDOCK (v. 3.0.2) server^51,52^ with the structures of the PgiC1 and PgiC2 monomers as input. The docking calculation was repeated three times, with consistent results. The docked hybrid complex was relaxed using Rosetta all-atom refinement.

The quality of the modelled dimer structures was evaluated by computing Z-scores^53,54^ with the ProSA web server^55^. The computed Z-scores ($-10.93$ for PgiC1 homodimer, $-10.83$ for PgiC2 homodimer, and $-10.85$ for the PgiC1–PgiC2 heterodimer) suggest a satisfactory quality of the modelled structures (Supplementary Fig. S3). The structural location of the variable residue sites, at which the analysed PgiC1 and PgiC2 sequences differ, and the structural effects of the mutations were examined using DeepView/Swiss-PdbViewer (v. 4.1.0)^56^. Predictions of the functional effect of the residue differences between the PgiC1 and PgiC2 sequences were computed using the SNAP2 program^24–26^ (Supplementary Table S1).

### Biophysical modelling

Under cellular conditions, proteins are not rigid bodies, but undergo structural fluctuations. Therefore, to better understand the biophysical properties of the three PgiC dimers, biomolecular simulations were conducted, using the MC program package PROFASI^57^. Two types of simulations were performed. One type explored the conformational ensembles sampled by the dimers in their native states, and the other investigated the ability of the dimers to withstand dissociation when subject to a stretching force. With >1000 residues, the systems have a computationally challenging size. However, systems of comparable size have been studied before with the same program^58^. Note also that it has been demonstrated that MC sampling can be a viable alternative to the more widely used molecular dynamics approach for dense protein systems^59^.

The PROFASI program combines an all-atom protein representation with an implicit solvent force field^31,60,61^. The degrees of freedom are the backbone Ramachandran torsion angles and side-chain torsion angles, while bond lengths, bond angles and peptide torsion angles are kept fixed^60,61^. A full description of the force field can be found elsewhere^31^. In brief, it is given by an interaction potential composed of four major terms: $E = E_\text {loc} + E_\text {ev} + E_\text {hb} + E_\text {sc}$^31^. Here, the first term, $E_\text {loc}$, describes local interactions between neighbouring peptide units along the polypeptide chains, while the other three terms represent non-local interactions. The terms $E_\text {ev}$ and $E_\text {hb}$ model steric repulsion and hydrogen bonding, respectively. Hydrogen bonding can occur between backbone CO and NH groups, and between charged side chains (Asp, Glu, Arg, Lys) and the backbone. The last term, $E_\text {sc}$, comprises pairwise sequence-dependent interactions between side chains, based on hydrophobicity and charge.

For the present study, an auxiliary constraint term, $E_\text {RMSD}$, was added to the above energy function *E*. This term is a penalty energy, which serves to statistically suppress large-scale fluctuations in the structure of the monomer units. The $E_\text {RMSD}$ energy is in turn composed of two terms, one for each monomer, which are proportional to the respective backbone RMSDs from the predicted monomer structures. Adding the $E_\text {RMSD}$ term stabilises the monomers near their predicted structures while allowing structural fluctuations as well as relative reorientations to occur in the simulated ensembles. Note that as this penalty depends only on the overall monomer RMSDs, structural fluctuations can occur anywhere along the polypeptide chains, both in the backbone and side-chain conformations, as well as in the relative organisation of the monomers.

All simulations were started with the dimer in a native-like conformation, derived from and similar to the homology-modelled structure. The temperature was set to 300 K. The simulation box was cubic, with a side length of 120 Å. Periodic boundary conditions were imposed.

The analysis of simulation data was carried out using STRIDE^27^ for secondary-structure assignments, FreeSASA^29^ for calculating solvent-accessible surface areas, PRODIGY^30^ for estimating free energies of binding, and YASARA^32^ for determining the abundance and nature of inter-monomer contacts.

Statistical comparisons of computed properties of the three studied dimers were done either with a one-way ANOVA test followed by Tukey’s range test, or with a Kruskal–Wallis test followed by a pairwise Wilcoxon signed ranks test (with correction for multiple testing) if the assumptions of homogeneous variances and/or normality for the ANOVA test were not fulfilled^28^. The statistical tests were performed using R.

### Native-state simulations

Using the biophysical model described above and MC sampling, the native-state ensembles of the dimers were explored. To gather statistics, a set of 24 independent runs was generated for each of the three dimers. Although no inter-monomer constraint was imposed, the dimers stayed intact throughout the runs. Each run comprised 500,000 MC cycles, which translates to about $2.5\times 10^9$ attempted elementary MC moves; one MC cycle corresponds to one attempted move per degree of freedom. The first 200,000 MC cycles of each run were discarded for equilibration. A total of $24\times 3,000$ conformations were stored for subsequent analysis for each dimer.

### Pulling simulations

As an independent way of assessing the tightness of the inter-monomer binding, an additional set of runs was performed in which the monomers were pulled apart by an external force. The external force was taken to act on the $\hbox {C}_\alpha$ atom located closest to the centre of mass of each monomer in the predicted native structure. The presence of the force was modelled by adding a term given by $F|\mathbf {x}_1-\mathbf {x}_2|$ to the energy function, where $\mathbf {x}_1$ and $\mathbf {x}_2$ are the central $\hbox {C}_\alpha$ atoms of the respective monomers and *F* denotes the strength of the force. The strength was set to $F=368$ pN and held constant. The inset of Fig. 4 illustrates the MC evolution of the system in a typical run. The run is started with the dimer in its native state, and the dimer remains native-like for a significant amount of time, until a sudden dissociation of its monomers occurs. The dissociation event is stochastic, so the time at which it occurs, $t_\text {dis}$, varies from run to run. To determine the average $t_\text {dis}$, a set of 24 runs was generated for each dimer, where each run comprised 500,000 MC cycles.

## Supplementary information

Supplementary information 1.

Supplementary information 2.
