# Supplementary material for: When a foreign gene meets its native counterpart: computational biophysics analysis of two PgiC loci in the grass Festuca ovina
Source: Sci Rep. 2020 Oct 30;10:18752. doi: 10.1038/s41598-020-75650-0 (PMC7599235; doi:10.1038/s41598-020-75650-0)
Supplement: Supplementary file 1 — Supplementary information 1. [file 41598_2020_75650_MOESM1_ESM.pdf]

Supplementary Information for: When a foreign gene meets its native counterpart: computational biophysics analysis of two *PgiC* loci in the grass *Festuca ovina*

Yuan Li[1], Sandipan Mohanty[2], Daniel Nilsson[1], Bengt Hansson[3], Kangshan Mao[4], Anders Irbäck[1]

[1]Computational Biology and Biological Physics, Department of Astronomy and Theoretical Physics, Lund University, SE-223 62, Lund, Sweden
[2]Institute for Advanced Simulation, Jülich Supercomputing Centre, Forschungszentrum Jülich, D-52425 Jülich, Germany
[3]Department of Biology, Lund University, SE-223 62, Lund, Sweden
[4]Key Laboratory of Bio-Resource and Eco-Environment of Ministry of Education, College of Life Sciences, State Key Laboratory of Hydraulics and Mountain River Engineering, Sichuan University, 610065, Chengdu, China

Table S1: **Protein sequence differences and basic characteristics of the variable sites**. List of the residue positions at which the protein sequences of *F. ovina* PgiC1 and PgiC2 differ (GenBank accession nos. AED99454 and AED99455). The list only includes positions at which both published sequences are well defined. For each of the 20 variable positions, three structural characteristics are listed: its secondary structure, the domain or region where it is found, and whether it is buried or near the surface of the dimer. The locations of the variable sites are indicated in the 3-D dimer structure shown in Figure S1. Also indicated in this figure are the small and large monomer domains and the N- and C-terminal regions. A site is defined as buried if the solvent accessible fraction of the surface area is $<15\%$. The last column shows predicted scores for the functional impact of single-residue PgiC1/PgiC2 mutations, as obtained using the program SNAP2 [1–3]. The score is between -100 and 100, where values >50 and <-50 respectively indicate effect and no effect of the mutation. Scores between -50 and 50 are ambiguous. The computed scores do not indicate effect for any of the 20 mutations, while indicating no effect for nine of them (shown in bold).

Pos.	PgiC1/PgiC2	Local structure	Domain/region	Buriedness	Comments	SNAP2
48	I/V	β-sheet	N-terminal	buried		**−86**
49	Y/F	β-sheet	N-terminal	surface		−8
53	S/A	loop	N-terminal	buried		**−56**
62	I/V	helix	N-terminal	buried		**−81**
85	N/D	loop	N-terminal	surface		−26
109	H/N	loop	N-terminal	surface		32
118	E/K	helix	small	surface		19
121	A/S	helix	small	surface		**−88**
123	I/K	helix	small	buried		−8
200	N/D	loop	small	surface	dimer interface	−46
210	I/V	β-sheet	small	buried		**−79**
237	H/P	loop	small	surface		42
266	G/A	β-sheet	small	surface		15
312	S/A	loop	none	surface		−39
318	L/I	helix	large	buried		**−85**
372	R/P	β-sheet	large	surface		35
455	L/V	loop	large	surface		**−59**
466	Q/K	loop	large	surface	dimer interface	**−53**
521	Q/R	helix	C-terminal	surface		**−61**
554	K/E	loop	C-terminal	surface		43

Figure S1: **Schematic illustration of the 3-D structure of the PgiC1 dimer**. One monomer is shown in yellow. For the other, a combination of colors is used, to indicate different parts of the molecule: the globular small (green) and large (orange) domains [4], the C-terminal (light blue) and N-terminal (purple) regions, and a helical inter-domain linker (white). The two catalytic centers, comprised of residues from both monomers (Glu360, His391, Lys516) [5], are shown in dark grey. Finally, the residues at the 20 variables sites (Tables S1) are shown in dark blue, with their position indices indicated.

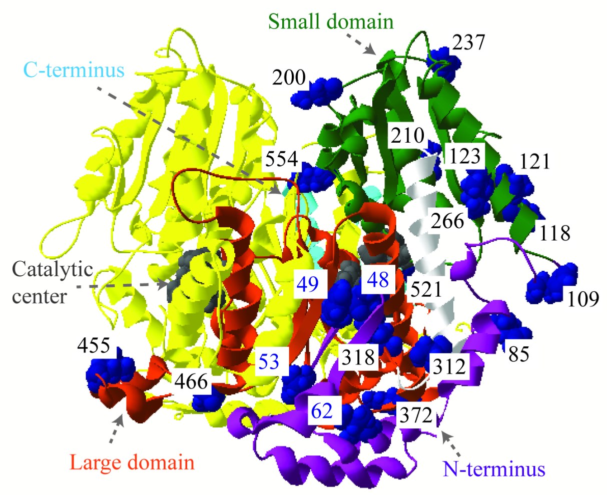

Figure S2: **Residue-specific secondary-structure probability profiles for the PgiC1 homodimer (black), the PgiC2 homodimer (green) and the heterodimer (red)**. The profiles were computed using the simulated native-state ensembles (see Main Text) and STRIDE [6] secondary-structure assignments. For each dimer, the data shown represent averages over its two chains. (a) Strand propensity. At positions 364, 377, 418 and 425, the β-strand propensity is notably higher for the PgiC2 homodimer than for the other systems. (b) Helix propensity.

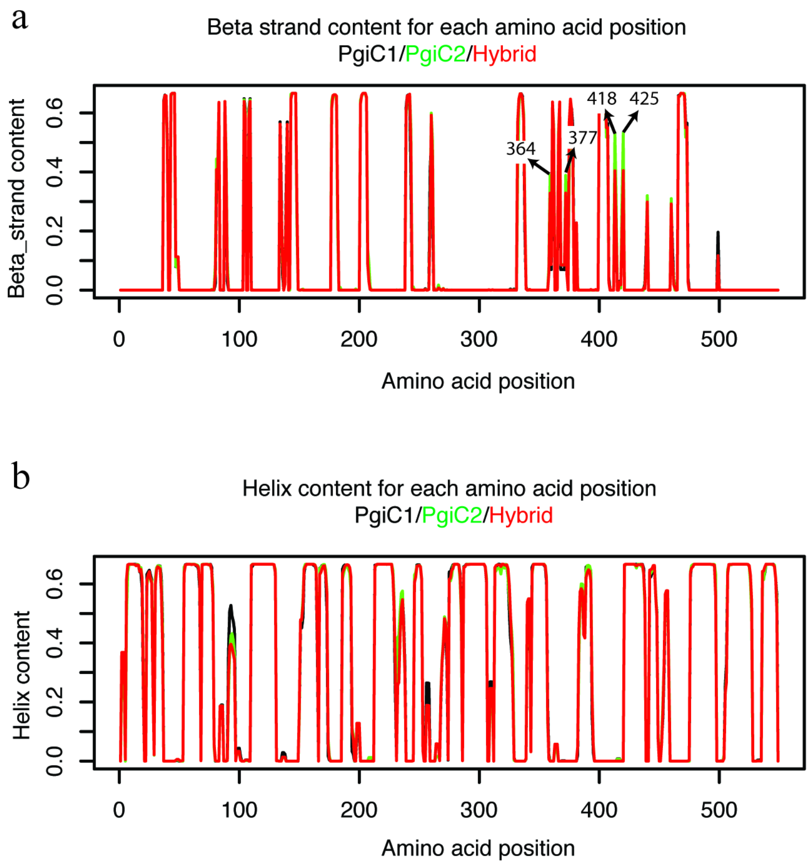

Figure S3: **Z-score assessment of the quality of the modelled protein structures**. Z-scores [7, 8] of the modelled native dimer structures (see Main Text) were computed using the ProSA web server [9] and are shown as black dots for (a) the PgiC1 homodimer, (b) the PgiC2 homodimer, and (c) the PgiC1-PgiC2 heterodimer. Blue dots are the same in all three panels and represent Z-scores for all structures in the Protein Data Bank (PDB) [10], determined by X-ray crystallography or NMR. The Z-scores of the modelled structures fall within the Z-score range of PDB structures with similar numbers of residues.

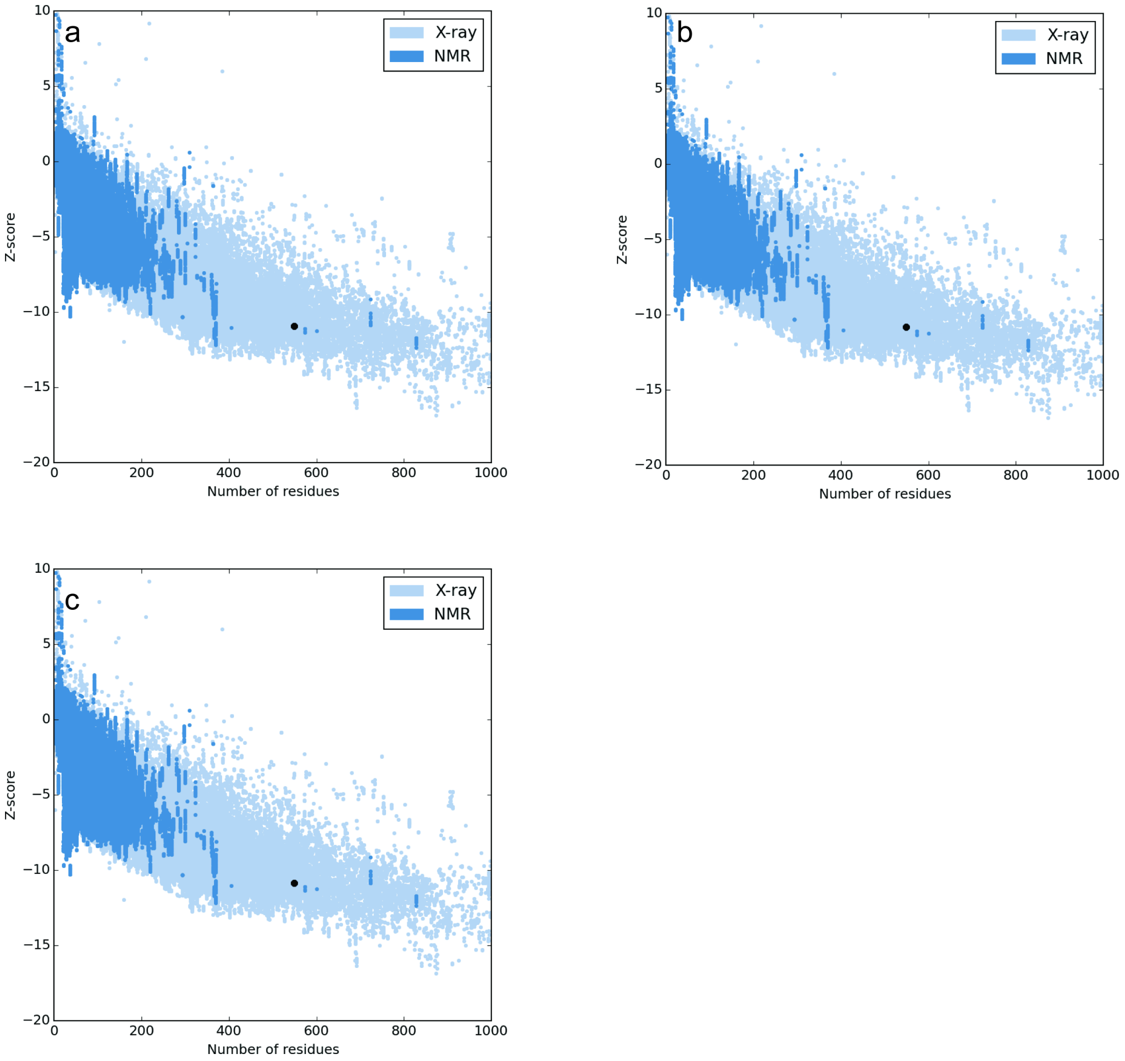

References

[1] Bromberg, Y. & Rost, B. SNAP: predict effect of non-synonymous polymorphisms on function. *Nucleic Acids Res.* **35**, 3823–3835 (2007).

[2] Hecht, M., Bromberg, Y. & Rost, B. News from the protein mutability landscape. *J. Mol. Biol.* **425**, 3937–3948 (2013).

[3] Hecht, M., Bromberg, Y. & Rost, B. Better prediction of functional effects for sequence variants. *BMC Genomics* **16**, S1 (2015).

[4] Wang, B., Watt, W. B., Aakre, C. & Hawthorne, N. Emergence of complex haplotypes from microevolutionary variation in sequence and structure of *colias* phosphoglucose isomerase. *J. Mol. Evol.* **68**, 433–447 (2009).

[5] Jeffery, C. J., Hardré, R. & Salmon, L. Crystal structure of rabbit phosphoglucose isomerase complexed with 5-phospho-D-arabinonate identifies the role of Glu357 in catalysis. *Biochemistry* **40**, 1560–1566 (2001).

[6] Heinig, M. & Frishman, D. STRIDE: a web server for secondary structure assignment from known atomic coordinates of proteins. *Nucleic Acids Res.* **32**, W500–W502 (2004).

[7] Sippl, M. J. Recognition of errors in three-dimensional structures of proteins. *Proteins* **17**, 355–362 (1993).

[8] Sippl, M. J. Knowledge-based potentials for proteins. *Curr. Opin. Struct. Biol.* **5**, 229–235 (1995).

[9] Wiederstein, M. & Sippl, M. J. ProSA-web: interactive web service for the recognition of errors in three-dimensional structures of proteins. *Nucleic Acids Res.* **35**, W407–W410 (2007).

[10] Berman, H. M., Westbrook, J., Feng, Z., Gilliland, G., Bhat, T., Weissig, H., Shindyalov, I. N. & Bourne, P. E. The Protein Data Bank. *Nucleic Acids Res.* **28**, 235–242 (2000).
